# Prior Adversity and Current Functioning Difficulties Predict Likelihood of Meeting the Criteria for Post-Traumatic Stress Disorder and Scoring Above the Cutoff for Post-Traumatic Growth

**DOI:** 10.3390/healthcare14101402

**Published:** 2026-05-20

**Authors:** Lourdes P. Dale, Audrey N. Dana, Kourtney L. Schroeder, Laren M. Alexander, Erin R. Heath, Stephen W. Porges, Steven P. Cuffe

**Affiliations:** 1 Department of Psychiatry, University of Florida College of Medicine—Jacksonville, Jacksonville, FL 32209, USA; 2 Department of Psychology, University of North Florida, Jacksonville, FL 32224, USA; 3 Traumatic Stress Research Consortium, Kinsey Institute, Indiana University, Bloomington, IN 47408, USA; 4 Department of Psychiatry, University of North Carolina at Chapel Hill, Chapel Hill, NC 27514, USA

**Keywords:** adversity, trauma, PTSD, post-traumatic growth, autonomic reactivity, negative world assumptions

## Abstract

Background/Objectives: Given that post-traumatic stress disorder (PTSD) and post-traumatic growth (PTG) are separate constructs that can co-occur following adversity, we examined how prior adversity and current functioning difficulties may relate to the likelihood of meeting criteria for PTSD and scoring above the cutoff for PTG among individuals who reported being impacted by their prior adversity. Methods: Participants (*n* = 2112) in this international sample completed online measures assessing their adversity history, current functioning difficulties (i.e., negative world assumptions and autonomic reactivity), PTSD symptomatology, and PTG. Results: Chi square analyses suggested a trend toward an association between meeting criteria for PTSD and scoring above the cutoff for PTG, although not statistically significant (*p* = 0.061). Multivariable logistic regression analysis found that individuals most impacted by caregiver abuse and certain specific adversities (i.e., parent with a mental health problem, caregiver and non-caregiver sexual abuse, and being held captive) were more likely to meet the criteria for PTSD. Whereas those most impacted by life-threatening situations and the specific adversities of being impacted by a life-threatening illnesses or injury, were more likely to meet the criteria for PTG. However, the strongest predictor of the likelihood of PTSD was increased autonomic reactivity, and the strongest predictor of the likelihood of PTG was fewer negative world assumptions. Conclusions: Our research suggests the need to assess the perceived impact of adversity history, as well as the potential negative consequences of autonomic reactivity and negative world assumptions, as these may be associated with PTSD symptomatology and PTG.

## 1. Introduction

Adversity refers to the full range of potential life stressors, which researchers have conceptualized to include experiences such as a health condition, life-threatening illness/injury, war exposure, natural disaster, captivity, physical/emotional/sexual abuse, or parental unavailability or mental/medical health problems [1]. Individuals with an adversity history may be resilient and not experience clinically significant difficulties. However, some individuals may experience negative outcomes, including mental health disorders [2], such as post-traumatic stress disorder (PTSD) [3].

There are various theories suggesting the factors that increase or decrease the likelihood of developing and maintaining PTSD symptoms. Cognitive-based theories suggest that when individuals experience traumatic events, they may experience cognitive dissonance that challenges their personal beliefs about oneself and the world, which may in turn lead to PTSD [4]. A specific cognitive theory, called the Shattered Assumptions theory, suggests that this occurs because the individual experiences the traumatic event as being inconsistent with their perception of the world as benevolent and meaningful [5,6]. Prior research has supported the belief that trauma may negatively impact people’s world assumptions, which may lead to PTSD symptoms [7,8]. Research also suggests that the shattered assumptions of safety may lead to PTSD symptoms that relate to physiological reactivity [5], such as hyperarousal and avoidance of trauma-related stimuli.

Polyvagal Theory (PVT) [9,10] provides a systems-level framework for understanding how adversity and chronic stress may influence autonomic regulation and contribute to PTSD-related symptoms. Within this framework, neuroception refers to implicit neural processes that evaluate cues of safety, danger, and life threat outside conscious awareness [11,12,13]. According to PVT, detection of danger may shift autonomic state toward defensive mobilization supported by sympathetic activation, commonly associated with fight-or-flight responses. Detection of life threat, or situations in which mobilization is ineffective, may shift autonomic state toward defensive immobilization mediated through dorsal vagal pathways. Although these autonomic responses function adaptively to support survival, persistent defensive states may compromise physiological flexibility, emotion regulation, bodily regulation, and capacity for social engagement [9]. Empirical studies have reported associations among adversity history, autonomic reactivity, and adverse mental health outcomes [1,14]. However, Polyvagal Theory should be understood as an interpretive framework for understanding autonomic regulation and adaptive behavioral responses rather than as a definitive explanatory model.

Regardless of whether they experience negative outcomes and/or PTSD, there is emerging evidence suggesting that individuals with history of adversity may experience post-traumatic growth (PTG) if they endured a certain level of trauma and were impacted by their experience [15,16]. Specifically, PTG refers to the positive changes that may occur with regard to five domains (i.e., relating to others, new possibilities, personal strength, spiritual change, and appreciation for life) [16]. Research suggests increased likelihood of self-reported PTG in individuals who have experienced sexual assault [17], intimate partner violence [18], and medical issues [19,20]. However, research has also found that self-report PTG is related to other constructs such as social desirability and dispositional optimism [21], as well as cognitive biases including downward comparison bias and positive attention bias [22]. Thus, it may be that these self-reported improvements are an illusion of growth rather than actual growth. Given these constraints, researchers caution that self-reported PTG may not represent the extent of actual change [23].

Thus, PTSD and PTG are unique responses to trauma that may co-occur in the same individual [24]. The relationship between them is complex, with prior research yielding inconsistent findings. Specifically, studies have suggested both a positively [25] and negatively correlated relationship [26], while others suggest the association may be curvilinear [27]. Therefore, it is important to conduct more research that investigates the relationship between these variables and how they relate to adversity history. This research needs to move beyond examining the occurrence of specific types of adversity, which may have occurred as single or multiple events, and to inquire about the perceived impact of these events.

Given that PTSD and PTG occur in response to adversity, we focused only on individuals who reported being impacted by at least one of 18 prior adverse experiences and indicated that their most adverse experience fell into one of five types (i.e., childhood adverse environment, caregiver abuse, non-caregiver abuse, life-threatening situations, or personal health situations). Because we asked which adversity type was the most impactful, we were able to create adversity type scores, which gives a higher score to the adversity type that was perceived as most impactful.

Given that prior research suggests an association between adversity history, people’s world assumptions [7,8], and autonomic reactivity [1,14], we also focused on these aspects of current functioning as potential predictors of PTSD and PTG. Thus, the current study sought to examine which of the five types of adversity and 18 specific adverse experiences were associated with increased likelihood of meeting criteria for PTSD and scoring above the cutoff for PTG, both individually and when examined together. The current study also explored whether the adversities identified as significant predictors would remain when considering current functioning difficulties (i.e., negative world assumptions and autonomic reactivity) and demographic characteristics (i.e., gender, age, and education level).

## 2. Materials and Method

### 2.1. Participant Recruitment and Procedure

Data collection for this online study began after the study’s protocol was approved by Indiana University’s Institutional Review Board (Ref. No.: 2003911917) on 22 March 2021. Participants were eligible to participate if they were 18 years or older. Online recruitment was primarily carried out via posts on Reddit, Twitter, Facebook, Instagram, and email lists. However, additional recruitment was carried out through Qualtrics Panels in the hopes of recruiting more participants who identify as male, non-Caucasian, and fall into a low socioeconomic status. Participants who were recruited via Qualtrics received payment in accordance with their chosen compensation plan (e.g., cash and airline miles).

Participants were presented with the consent form at the start of the survey, which was completed via Qualtrics XM. Those that consented to participate were presented with the measures to complete. Automated quality checks and manual inspection were used to ensure all responses were high quality. Responses were flagged, reviewed, and possibly removed if they contained duplicated information and were completed faster than 25% of the median completion time. Following this procedure, the data set included 3627 participants. After additional participants (*n* = 1515) were dropped because they did not report being impacted by any adversity or had incomplete data for the primary measures in the study (e.g., autonomic reactivity, PTSD, and PTG), the remaining sample included 2112 participants.

### 2.2. Measures

Demographic data (i.e., gender, racial identity, age, and education) were collected. Below is a description of the study constructs and measures. 

#### 2.2.1. Adversity History Variables

Participants initially completed an earlier version of the Adverse and Traumatic Experiences Scale (ATES) [28], which asks participants to identify the impact of various adversities via a 4-point Likert scale (0 = *event did not occur*, 1 = *event occurred and had a minimal impact*, 2 = *event occurred and had some impact*, and 3 = *event occurred and had a big impact on my life*). The current study focused on the 18 items related to the following subscales: Childhood Adverse Environment (6 items: unavailable parent, parents divorced, parent with substance use problem, parent with severe medical illness, parent with mental health issues, parent was physically, sexually, or emotionally abused); Caregiver Abuse (3 items: emotional abuse, physical abuse, sexual abuse); Non-caregiver Abuse (2 items: physical abuse, sexual abuse); Life-Threatening Situations (4 items: experiencing a natural disaster, war zone, held captive, witnessing a serious injury); and Personal Health Problems (3 items: chronic health conditions, life-threatening illness, life-threatening injury).

Following the ATES, participants were asked to consider their reported adversity history and to identify which type of experience was the most impactful (e.g., life-threatening situation, parental/caregiver adverse experience, or childhood abuse) or to write in a different type of adverse experience. Many individuals wrote in responses that related to non-caregiver abuse and personal health problems.

In the current study, participant scores for each of the 18 items were recoded to create specific adversity experience scores, which were coded as 0 if the event did not occur or had no impact and as 1 if the event occurred and had a moderate or large impact. These specific adversity scores were used to determine if the participants were impacted by at least one of the specific adversities in each of the five adversity types. The reported information of being impacted by a specific adversity was combined with the endorsed reports about the most impactful experience to create the five adversity type scores, which represents whether the individual was not impacted (scored as 0), impacted (scored as 1), or impacted and viewed this experience as most impactful (scored as 2). The decision to combine the data from two variables (i.e., the variable focused on whether they were impacted by each adversity type and the variable that indicated which adversity was the most impactful) was made because of the overlap in the information provided, as it can be assumed that individuals were impacted by their identified most impactful experience. In addition, this allowed us to contribute to the field by assessing whether the most impactful experience was more predictive of later functioning than just being impacted by an experience.

#### 2.2.2. Outcomes

PTSD symptoms were assessed via the 17-item PTSD Checklist Civilian Version [29], a measure used to evaluate distress levels within the last month. This internally consistent measure (*α* = 0.95) asks participants to identify their level of distress via a 5-point Likert scale (0 = *not all* to 4 = *extremely*). The current study focused on whether or not the individual reported sufficient symptoms to meet DSM-IV criteria for PTSD by first treating responses reported to be 2–4 (Moderately or above) as symptomatic and responses 1–2 (below Moderately) as non-symptomatic. Next, individuals were coded as meeting the criteria if they were symptomatic for at least one criteria B item (Questions 1–5), at least three criteria C items (Questions 6–12), and at least two criteria D items (Questions 13–17).

PTG was assessed via the 21-item Post-traumatic Growth Inventory [16], which asks respondents to indicate the degree to which change occurred after a traumatic crisis using a 6-point Likert scale (0 = *I did not experience this change as a result of my crisis* to 5 = *I experienced this change to a very great degree as a result of my crisis*). The internally consistent measure (*α* = 0.95) includes five scales (i.e., Appreciation for Life, Spiritual Change, Relationship with Others, Personal Strength, and New Possibilities). Similar to prior research [30], we used a total score of 46 or higher to indicate PTG, so we could compare individuals who did and did not report PTG.

Negative world assumptions were assessed via the 22-item World Assumptions Questionnaire [31], which includes items related to comprehensibility and predictability of people, trustworthiness/goodness of people, controllability of events, and safety/vulnerability. Sample items include: (1) Most people can be trusted; (2) I don’t feel in control of the events that happen to me; and (3) People are less safe than they usually realize. Respondents indicate their level of agreement with assumptions via a 6-point Likert scale, which in the current study was reversed so higher scores would indicate more negative world assumptions (1 = *strongly disagree* to 6 = *strongly agree*). We also combined the scores for all items to create internally consistent (*α* = 0.88) negative world assumptions scores.

Autonomic reactivity was measured via the Body Perception Questionnaire Short Form [32,33,34]. This 20-item measure asks about experiences of reactivity and coordination in organs and tissues regulated by the autonomic nervous system. Sample questions include: (1) *I have difficulty coordinating breathing and eating*; (2) *I gag from the saliva in my mouth*; and (3) *When I breathe, I feel like I cannot get enough oxygen*. The respondent indicates frequency of specific bodily sensations via a 5-point Likert scale (1 = *never* to 5 = *always*). Higher scores indicate greater autonomic reactivity, which prior research found to be associated with higher resting heart rate, lower parasympathetic activity, and less parasympathetic/sympathetic flexibility in response to challenges [35]. Prior research suggests that this internally consistent self-report measure of autonomic reactivity has a consistent factor structure across samples, along with convergent validity and high test-retest reliability [32,36,37].

### 2.3. Data Analysis

Data analysis was conducted using IBM SPSS Statistics for Windows, Version 29.0 (IBM Corp., Armonk, NY, USA). The current manuscript uses an alpha cutoff of 0.05 for statistical significance. Descriptive statistics were run to understand the participants with regard to their demographic characteristics (e.g., gender, race, and education level), adversity history, and current functioning difficulties. Chi square analyses explored the association between likelihood of meeting the criteria for PTSD and PTG.

Prior to running logistic regression analyses, *z*-scores were calculated for the linear variables (i.e., negative world assumptions, autonomic reactivity, and age) so that odds ratios would represent standard deviation changes and be more easily compared across these variables. Next, univariable logistic analyses determined which measures of adversity history and current functioning difficulties were predictive of PTSD and PTG. Separate multivariable stepwise logistic regression analyses using forward conditional selection determined which variables were most predictive of PTSD and PTG. To address multicollinearity among the adversity types and specific adversity experiences, all the adversity variables (i.e., adversity types and the specific adversity experiences) were entered in Block 1. The current functioning variables were entered in Block 2, and the demographic variables (i.e., gender, age, and education level) were entered in Block 3.

## 3. Results

### 3.1. Description of Participants

The current manuscript focuses on individuals who were able to identify the adverse experiences that they viewed as most impactful within one of the five types that are the focus of this study (i.e., childhood adverse environment, caregiver abuse, non-caregiver abuse, life-threatening situations, and personal health situations). Participants (*n* = 2112), who primarily lived in the U.S. (62.17%), Europe (20.17%), and Canada (9.38%), varied in age from 18 to 88 years old (*M* = 47.28, *SD* = 14.35). Most participants reported identifying as female (78.65%) and Caucasian (48.72%), and having earned a graduate (55.82%) or bachelor’s degree (34.23%).

### 3.2. Adversity History and Current Functioning Difficulties

**Figure 1:**
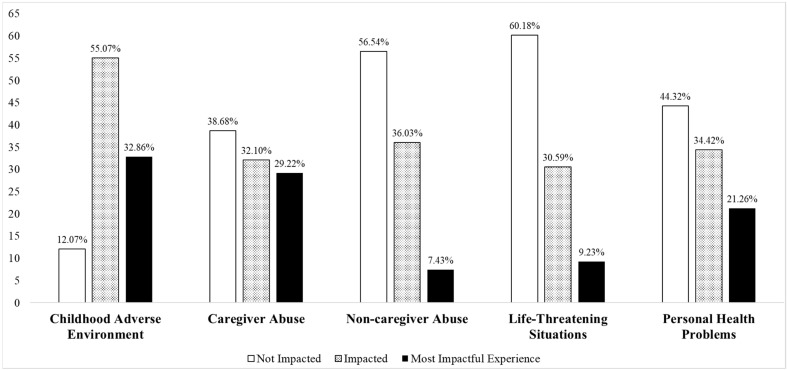
Reported adversity history. For each adversity type (i.e., childhood adverse environment, caregiver abuse, non-caregiver abuse, life-threatening situations, and personal health problems), the bar graph displays the percentage of participants who reported that the respective adversity type was not impactful, impactful, or the most impactful adversity type experienced.

Figure 1 displays the percent of individuals that fell into each of the categories for the adversity types (i.e., not impacted, impacted, and most impactful life experience). Examination of the percentages indicates that few participants were not impacted by at least one of the six childhood adverse environments (12.07%) and/or caregiver abuse (38.68%). For most impactful experience, the highest percentages reported were related to the childhood adverse environment (32.86%) and caregiver abuse (29.22%). Fewer individuals reported being most impacted by non-caregiver abuse (7.43%), life-threatening situations (9.23%), and personal health problems (21.26%). With regard to the specific adversities, Table 1 documents the highest percentages of being impacted were found with regard to caregiver emotional abuse (56.11%), parent was abused (50.14%), parent with serious mental health problems (45.93%), and chronic health condition (42.71%).

There was variability regarding current functioning difficulties. Negative world assumptions total scores ranged from 24 to 114 (*M* = 73.20, *SD* = 14.57), and z-scores ranged from −2.80 to 3.38. Autonomic reactivity total scores ranged from 33 to 83 (*M* = 50.12, *SD* = 9.45), with z-scores ranging from −1.79 to 3.53. Pearson correlational analyses indicated that these measures of current functioning difficulties were correlated with each other (*r* = 0.28, *p* < 0.001).

### 3.3. Predicting PTSD and PTG

As expected, given the focus on individuals who were impacted by at least one adversity, 31.30% of individuals reported symptoms consistent with the DSM-IV diagnostic criteria for PTSD and 62.97% of individuals met the criteria for PTG. We found that 18.80% of participants met the criteria for both PTSD and PTG. Although chi square analysis, which examined whether individuals who met the criteria for one also met the criteria for the other, did not reach statistical significance, χ^2^ (*n* = 2112) = 3.50, *p* = 0.061, there was a trend suggesting a potential association.

#### 3.3.1. Predicting PTSD

**Figure 2:**
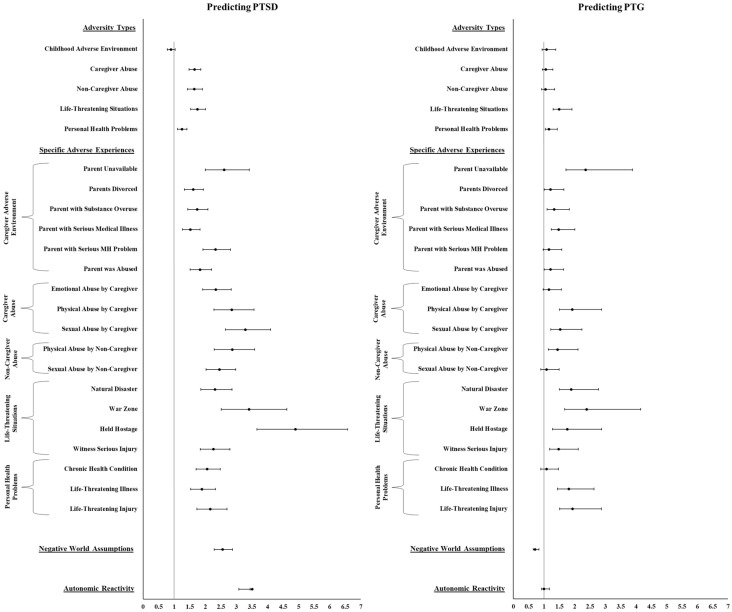
Univariable logistic regression predicting post-traumatic stress disorder (PTSD) and post-traumatic growth (PTG). Forest plot displaying odds ratios and 95% confidence intervals from univariable logistic regression analyses predicting likelihood of PTSD and PTG.

As reported in Table 2 and Figure 2, univariable logistic regression analyses found that all the adversity types, specific adversities, and current functioning difficulties (i.e., negative world assumptions and autonomic reactivity) were associated with increased likelihood of meeting criteria for PTSD. Table 2 also indicates that the demographic characteristics examined were associated with likelihood of meeting criteria for PTSD. Specifically, there was an increased likelihood for individuals who reported being male, younger, and less educated.

As reported in Table 3, multivariable logistic regression analyses which considered the adversity types and the specific adversities in Block 1 suggested that the significant predictors were only adversity type, specifically caregiver abuse, and the following specific adversities: parent with mental health issues, caregiver sexual abuse, non-caregiver sexual abuse, held captive, natural disaster, chronic health condition, and life-threatening injury. When the current functioning variables were entered as potential predictors, autonomic reactivity was selected as the next significant predictor, followed by negative world assumptions. Only four specific adverse experiences (i.e., parent with mental health issues, non-caregiver sexual abuse, natural disaster, and life-threatening injury) remained as significant predictors when the current functioning variables were included as predictors. Younger age emerged as the only demographic factor that was predictive of PTSD in Block 3, and it changed the predictiveness of two specific adversity variables. Specifically, chronic health conditions re-emerged as a significant predictor and the predictiveness of non-caregiver sexual abuse was diminished.

The final model, which correctly classified 79.5% of participants, suggested that the strongest predictors were the measures of current functioning difficulties, with autonomic reactivity being a stronger predictor than negative world assumptions. Examination of the odds ratios indicates that individuals scoring 1 standard deviation above the mean with regard to autonomic reactivity were 2.3 times more likely to meet the criteria for PTSD, whereas those scoring 2 standard deviations above the mean were 5.3 times more likely to meet the criteria. Similarly, individuals scoring 1 standard deviation above the mean with regard to negative world assumptions were 2.2 times more likely to meet the criteria for PTSD, whereas those scoring 2 standard deviations above the mean were 4.8 times more likely to meet the criteria. The current functioning variables were stronger predictors than the most predictive measure of prior adversity, as the next strongest (i.e., parent with a mental health problem) resulted in 1.6 times greater risk of meeting the criteria for PTSD.

#### 3.3.2. Predicting PTG

As reported in Table 2 and Figure 2, univariable logistic regression analyses indicated that life-threatening situation was the only adversity type that was associated with greater odds of meeting the criteria for PTG. Consistent with this result, the odds were greater for individuals impacted by all four life-threatening situations (i.e., natural disaster, war zone, held hostage, and witnessed serious injury). Other specific adversity experiences that were predictive were: parent unavailable, parents divorced, parent with a substance use problem, parent medically ill, parent was abused, caregiver physical and sexual abuse, non-caregiver physical abuse, and life-threatening illness and injury. In addition, individuals were more likely to meet the criteria for PTG if they reported fewer negative world assumptions. Table 2 also indicates that older age was the demographic characteristics examined that was associated with likelihood of scoring above the cutoff for PTG.

As reported in Table 4, multivariable logistic regression analyses which considered the adversity types and the specific adversities in Block 1 suggested that the significant predictors were only adversity type, specifically life-threatening situations, and the following specific adversities: parent unavailable, parent with medical illness, caregiver physical abuse, life-threatening illness, and life-threatening injury. These adversity history predictors remained significant predictors when the current functioning variable of negative world assumptions was selected as a predictor in Block 2 and when older age was selected as a predictor in Block 3.

The final model, which correctly classified 65.5% of participants, indicated that the specific adversity experience that was the strongest predictor was having an unavailable parent. Specifically, odds ratios indicated that individuals impacted by this experience were 1.6 times more likely to meet the criteria for PTG. Similar odds were found for individuals who reported life-threatening situations to be their most impactful experience. Although individuals impacted by this experience were 1.4 times more likely to meet the criteria, those that viewed it as the most impactful experience were 2.0 (i.e., 1.4 multiplied by 1.4 = 2.0) times more likely to meet the criteria for PTG. The negative world assumptions odds ratio of 0.69 indicates a reduction by 31.00% in likelihood of meeting the criteria for PTG with each standard deviation increase in negative world assumptions.

## 4. Discussion

Our study sought to understand how prior adversity and current functioning difficulties, as indicated by negative world assumptions and self-reported autonomic reactivity, may relate to the likelihood of scoring above the cutoff for post-traumatic stress disorder (PTSD) and post-traumatic growth (PTG). Given the belief that individuals need to be impacted by an adversity to develop PTSD and/or experience PTG, we limited this sample to only include the 2112 individuals who reported being impacted by at least one of the 18 specific life experiences and identified their most impactful adversity as being one of five adversity types.

Individuals in the current sample reported being most impacted by their childhood adverse environment (32.86%), caregiver abuse (29.22%), personal health problems (21.26%), non-caregiver abuse (7.43%), and life-threatening situations (9.23%). In addition, 31.30% of individuals met the DSM-IV diagnostic criteria for PTSD and 62.97% of individuals scored above the cutoff for PTG. The high rate of PTG is consistent with prior research suggesting that individuals with adversity histories may experience PTG if they endured a certain level of trauma and were impacted by their experience [15,16].

Our results are consistent with prior research suggesting that PTSD and PTG are unique responses to trauma that may co-occur in the same individual [24]. Specifically, our results suggested a weak association between these constructs, as likelihood of meeting the criteria for one was not statistically associated with greater likelihood of meeting the criteria for the other. We also found that likelihood of PTSD and PTG was associated with different measures of prior adversity history, current functioning difficulties, and the demographic variable of age when they were entered into multivariable logistic regression analyses.

As stated earlier, most individuals reported that their most impactful adverse experience occurred in their childhood (62.08%) in the form of childhood adverse environment or caregiver abuse. Although both childhood experiences were identified as most impactful, they differed in their relationship with the likelihood of meeting the criteria for PTSD. The overall findings suggest that clinicians should be cautious when aggregating childhood experiences to derive an Adverse Childhood Experiences (ACES) score, as the different types of adversities and experiences may be linked to different outcomes.

We found that individuals most impacted by caregiver abuse and the specific experience of having a parent with mental health issues and caregiver sexual abuse were more likely to meet the criteria for PTSD. In addition, we found that individuals that were impacted by the experience of having an unavailable parent, parent with a medical illness, and/or being physically abused by a caregiver were more likely to meet criteria for PTG.

Given prior research [38], it is not surprising that being impacted by the adverse childhood experience of having a parent with a serious mental health problem would be associated with increased likelihood of meeting the criteria for PTSD. However, it is surprising to find that being impacted by the experiences of having a parent that is unavailable and/or physically abusive would be associated with PTG.

Similar to the individuals impacted by caregiver sexual abuse, those impacted by non-caregiver sexual abuse were more likely to meet the criteria for PTSD. Thus, we found that the experience of sexual abuse, whether perpetrated by a caregiver or another individual, was associated with an increased likelihood of meeting the criteria for PTSD, a finding that is consistent with prior research [39,40]. This finding is particularly striking given the overlap, as we found that 67.29% of individuals who reported being impacted by caregiver sexual abuse also reported being impacted by non-caregiver sexual abuse. In addition, our finding is inconsistent with prior research that suggests greater PTG in individuals who have experienced sexual assault [17] and intimate partner violence [18].

With respect to life-threatening situations, we found that the likelihood of PTSD was higher for the individuals impacted by the specific adversity experiences of a natural disaster and being held hostage. In addition, the likelihood of PTG increased for individuals who reported being most impacted by a life-threatening situation, which includes natural disaster, being in a war zone, being held hostage, and witnessing serious injury. While this is consistent with prior research suggesting that survivors of natural disasters [41,42], veterans who experienced combat [43], and prisoners of war [44,45] display PTG, less is known about PTG after witnessing serious injury.

Regarding personal health problems, we found that individuals impacted by their life-threatening injury were more likely to meet the criteria for both PTSD and PTG. Whereas, individuals impacted by chronic health conditions were more likely to meet the criteria for PTSD. In contrast, individuals impacted by a life-threatening illness were more likely to meet the criteria for PTG, a finding that is consistent with the results of prior research suggesting an increased likelihood of PTG in individuals with medical issues [19,20], especially if it is life-threatening [46].

Although the prior adversities remained important predictors of the likelihood of PTSD, we found that the strongest predictor of the likelihood of meeting the criteria for PTSD was self-reported autonomic reactivity followed by negative world assumptions, which diminished the predictiveness of caregiver abuse and the specific experiences of caregiver sexual abuse, held captive, life-threatening injury, and chronic health conditions. This finding is consistent with the assumptions of polyvagal theory [9,10] and research findings [1,14] suggesting association between adversity history, autonomic reactivity, and negative mental health consequences. These findings are also consistent with prior research suggesting that trauma may negatively impact people’s world assumptions, which may in turn lead to PTSD symptoms [7,8]. In addition, we found that fewer negative world assumptions was the strongest predictor of likelihood to experience PTG, a finding that is somewhat unique as we found limited research linking these constructs [47,48]. Thus, it may be that both autonomic reactivity and negative world assumptions are associated with the development of PTSD, whereas only decreased negative world assumptions is associated with the development PTG.

## 5. Strengths and Limitations

The current study has major limitations relating to the data collection and the characteristics of the sample. To ensure a large international sample, participants were recruited through Reddit, Twitter/X, Facebook, Instagram, email lists, and Qualtrics Panels, with targeted panel supplementation to increase representation of men, non-Caucasian participants, and lower-SES participants. Although this recruitment process produced a large sample of individuals, the final analytic sample remained predominantly female, largely Caucasian, and highly educated, with most participants residing in the U.S., Europe, and Canada. This creates a substantial risk of selection bias related to internet access, self-selection into trauma-related research, education, and likely symptom salience. To address this limitation, we conducted analyses controlling for these demographic differences and found that only age emerged as a significant predictor of PTSD and PTG. However, this overrepresentation limits the generalizability of the findings. Future studies need to ensure that the recruitment procedures yield a more representative sample of the general population.

Another selection bias relates to the study’s inclusion of only individuals who reported being impacted by at least one adversity and who could identify one adversity type as most impactful. This substantially narrowed the inferential scope of the study, as the study does not address adversity-exposed individuals in general and those who do not perceive themselves as being impacted even though they may have been. The decision to focus on a selected subgroup of adversity-exposed individuals who already appraise adversity as impactful and can identify a dominant adversity category was made because of the belief that individuals need to be impacted by at least one adversity to be able to experience PTSD and/or PTG.

The results may have been influenced and limited by our scoring decisions. With respect to adversity history, participant scores for each of the 18 items were used to determine if the participant was impacted by at least one of the specific adversities in each of the five adversity types. This information was combined with the information about the most impactful experience to create a 3-point coding (i.e., 0 = adversity type did not occur or was not impactful, 1 = adversity type was impactful, and 2 = adversity type was the most impactful). Although this approach led to a combined variable that eliminated the overlap that would occur if the variable was looked at separately, different results may have been found if the variables had been examined separately.

The current study is also limited due to its reliance on online self-report measures, which may be impacted by various factors such as desirability. Additional concerns relate to our measurement of PTSD symptomatology, as the PCL-C is based on the diagnostic criteria for PTSD in the DSM-IV rather than the current DSM-5. The diagnostic differences (e.g., inclusion of another symptom cluster in the DSM-5) may have impacted the likelihood of meeting the diagnostic criteria of PTSD and in turn the results of this study.

To allow for consistency in results reporting, we used an established cutoff to dichotomize PTG scores. While this facilitated the grouping of individuals into those that did and did not report experiencing PTG, this may have been an oversimplification as PTG is generally understood as a multidimensional and often continuous construct. In addition, our results were limited by our decision to not focus on the PTG domains, such as appreciation of life, personal strength, or relational growth. This decision was made out of concern about presenting too many variables, as the manuscript focuses on various adversities, two current functioning variables, and the outcomes of PTSD and PTG. It is recommended that future research consider the multidimensionality of PTG as a continuous construct.

The current study measured autonomic regulation via a self-report measure of autonomic reactivity called the Body Perception Questionnaire [32,33,34]. Although this measure has been found to have strong internal consistency and convergent validity with laboratory measures of heart rate variability (i.e., heart period and respiratory sinus arrhythmia) [35], it does not provide the depth of information that can be obtained through heart rate data collection. For example, our measure does not provide information related to changes in autonomic regulation in response to stressors or relaxing experiences.

To address potential problems with the quality of the data, quality checks were employed to identify any possible invalid submissions (e.g., duplicate responses and very quick completion of the measures). In addition to self-report measures, future studies should include objective measures, such as biological and physiological markers of current functioning. For example, it would be important to know if the reported differences in self-reported autonomic reactivity are supported by differences in measures of heart rate variability.

A final limitation relates to the cross-sectional nature of the study. Although our findings provided important insights into the relationships between adversity history, current functioning difficulties, demographics, and PTSD and PTG, longitudinal research is needed to establish causal pathways and temporal ordering among these variables.

## 6. Conclusions

Our results suggest that individuals impacted by their adversity history may experience both negative and positive consequences. We found that individuals were more likely to meet the criteria for PTSD if they reported being most impacted by caregiver abuse, and the specific adversities of having a parent with mental health issues, experiencing caregiver and non-caregiver sexual abuse, experiencing a natural disaster, being held captive, having a chronic health condition, and experiencing a life-threatening injury. In contrast, we found that individuals were more likely to meet the criteria for PTG if they reported being most impacted by a life-threatening situation, and the specific adversities of having a parent who was unavailable, had a medical illness, or was physically abusive and experienced a life-threatening illness or injury. Lastly, we found that it was important to consider current functioning difficulties, as increased self-reported autonomic reactivity and negative world assumptions were strong predictors of likelihood of PTSD, whereas fewer negative world assumptions was the strongest predictor of likelihood of PTG.

Our research suggests the need for clinicians to provide their patients with measures, such as the Adverse and Traumatic Experiences Scale [28], to assess their perception of the impact of their prior adversities. They should also ask about the most impactful experience, as it was found to be statistically and clinically relevant, especially with respect to caregiver abuse and life-threatening situations.

Because our study found that significant increases in self-reported autonomic reactivity was associated with increased likelihood of meeting the criteria for PTSD, clinicians should determine if their patients are reporting heightened autonomic reactivity. In addition, clinicians should assess for the potential positive consequences that may have arisen from the prior adversities through measures of PTG, such as the Post-Traumatic Growth Inventory [16]. This information could be useful in tailoring the treatment to focus on cultivating growth from prior adversity, rather than focusing exclusively on attempting to decrease the negative consequences of adversity, such as PTSD symptoms. By assessing PTG periodically throughout the course of therapy, patients can reflect on their PTG over time and feel accomplished about their change and growth.

## Figures and Tables

**Table 1:** Reported adversity history.

Specific Adversity Experiences	Not Impacted	Impacted
	%	%
**Childhood Adverse Environment**		
Parent Unavailable	88.26	11.74
Parents Divorced	61.36	38.64
Parent with Substance Overuse	63.54	36.46
Parent with Serious Medical Illness	50.00	50.00
Parent with Serious MH Problem	54.07	45.93
Parent was Abused	49.86	50.14
**Caregiver Abuse**		
Emotional Abuse by Caregiver	43.89	56.11
Physical Abuse by Caregiver	81.25	18.75
Sexual Abuse by Caregiver	79.73	20.27
**Non-Caregiver Abuse**		
Physical Abuse by Non-Caregiver	81.30	18.70
Sexual Abuse by Non-Caregiver	66.00	34.00
**Life-Threatening Situations**		
Natural Disaster	78.74	21.26
War Zone	90.91	9.09
Held Hostage	89.49	10.51
Witness Serious Injury	76.70	23.30
**Personal Health Problems**		
Chronic Health Condition	57.29	42.71
Life-Threatening Illness	77.32	22.68
Life-Threatening Injury	80.54	19.46

MH = Mental Health.

**Table 2:** Univariable logistic regression predicting post-traumatic stress disorder (PTSD) and post-traumatic growth (PTG).

Variables	Predicting PTSD		Predicting PTG	
	*OR*	95% CI	*OR*	95% CI
**Adversity Types**				
Childhood Adverse Environment	0.90	0.78 to 1.03	1.08	0.94 to 1.24
Caregiver Abuse	1.66 ***	1.48 to 1.86	1.06	0.95 to 1.18
Non-Caregiver Abuse	1.65 ***	1.43 to 1.91	1.05	0.92 to 1.22
Life-Threatening Situations	1.75 ***	1.53 to 2.01	1.49 ***	1.30 to 1.72
Personal Health Problems	1.25 **	1.11 to 1.41	1.16 *	1.04 to 1.31
**Specific Adverse Experiences**				
**Childhood Adverse Environment**				
Parent Unavailable	2.61 ***	2.00 to 3.42	2.36 ***	1.72 to 3.25
Parents Divorced	1.61 ***	1.33 to 1.94	1.21 *	1.01 to 1.45
Parent with Substance Overuse	1.74 ***	1.44 to 2.09	1.33 **	1.10 to 1.60
Parent with Serious Medical Illness	1.52 ***	1.27 to 1.83	1.48 ***	1.24 to 1.77
Parent with Serious MH Problem	2.33 ***	1.93 to 2.81	1.16	0.97 to 1.39
Parent was Abused	1.83 ***	1.51 to 2.20	1.21 *	1.01 to 1.44
**Caregiver Abuse**				
Emotional Abuse by Caregiver	2.34 ***	1.92 to 2.84	1.16	0.97 to 1.38
Physical Abuse by Caregiver	2.85 ***	2.28 to 3.57	1.92 ***	1.50 to 2.46
Sexual Abuse by Caregiver	3.29 ***	2.65 to 4.10	1.53 ***	1.22 to 1.93
**Non-Caregiver Abuse**				
Physical Abuse by Non-Caregiver	2.87 ***	2.29 to 3.59	1.44 **	1.14 to 1.82
Sexual Abuse by Non-Caregiver	2.46 ***	2.03 to 2.98	1.08	0.90 to 1.31
**Life-Threatening Situation**				
Natural Disaster	2.31 ***	1.86 to 2.86	1.89 ***	1.50 to 2.39
War Zone	3.41 ***	2.52 to 4.62	2.39 ***	1.67 to 3.43
Held Hostage	4.90 ***	3.66 to 6.58	1.76 ***	1.28 to 2.40
Witness Serious Injury	2.26 ***	1.84 to 2.79	1.48 ***	1.19 to 1.83
**Personal Health Problems**				
Chronic Health Condition	2.06 ***	1.71 to 2.48	1.08	0.90 to 1.29
Life-Threatening Illness	1.89 ***	1.53 to 2.33	1.81 ***	1.44 to 2.26
Life-Threatening Injury	2.16 ***	1.73 to 2.70	1.93 ***	1.51 to 2.45
**Current Functioning Difficulties**				
Negative World Assumptions	2.56 ***	2.29 to 2.88	0.71 ***	0.65 to 0.78
Autonomic Reactivity	3.51 ***	3.08 to 4.01	1.00	0.92 to 1.10
**Demographic Characteristics**				
Female Gender	0.65 ***	0.52 to 0.80	0.86	0.69 to 1.07
Age	0.97 ***	0.96 to 0.97	1.01 **	1.00 to 1.02
Education Level	0.67 ***	0.58 to 0.77	1.12	0.98 to 1.28

* *p* < 0.05, ** *p* < 0.01, *** *p* < 0.001. MH = Mental Health.

**Table 3:** Multivariable logistic regression predicting post-traumatic stress disorder (PTSD).

Selected Predictors	OR (95% CI)	OR (95% CI)	OR (95% CI)
**Block 1**			
**Adversity Types**			
Caregiver Abuse	1.30 *** (1.12–1.50)	1.16 (0.99–1.37)	1.16 (0.98–1.37)
**Specific Adversities**			
Chronic Health Condition	1.74 *** (1.41–2.15)	1.27 (1.00–1.62)	1.44 ** (1.13–1.85)
Life-Threating Injury	1.36 * (1.04–1.77)	1.39 * (1.02–1.89)	1.40 * (1.03–1.91)
Natural Disaster	1.74 *** (1.36–2.24)	1.45 * (1.09–1.93)	1.47 ** (1.10–1.97)
Held Captive	2.39 *** (1.68–3.38)	1.52 (1.00–2.30)	1.42 (0.93–2.18)
Parent with Serious MH Problem	1.75 *** (1.41–2.18)	1.54 *** (1.20–1.97)	1.57 *** (1.22–2.02)
Caregiver Sexual Abuse	1.56 ** (1.18–2.05)	1.21 (0.88–1.66)	1.29 (0.94–1.78)
Non-Caregiver Sexual Abuse	1.45 ** (1.15–1.83)	1.31 * (1.01–1.70)	1.29 (0.99–1.68)
**Block 2**			
Autonomic Reactivity		2.54 *** (2.20–2.94)	2.34 *** (2.02–2.71)
Negative World Assumptions		2.23 *** (1.95–2.56)	2.21 *** (1.92–2.53)
**Block 3**			
Age			0.98 *** (0.97–0.98)
***df***	1, 8	2, 10	1, 11
**χ^2^**	308.60 ***	721.71 ***	753.68 ***

* *p* < 0.05, ** *p* < 0.01, *** *p* < 0.001. MH = Mental Health. The final model correctly classified 79.5% of participants.

**Table 4:** Multivariable logistic regression predicting post-traumatic growth (PTG).

Selected Predictors	*OR* (95% CI)	*OR* (95% CI)	*OR* (95% CI)
**Block 1**			
**Adversity Types**			
Life-Threatening Situations	1.29 ** (1.10–1.50)	1.36 *** (1.16–1.60)	1.37 *** (1.17–1.60)
**Specific Adversity Experiences**			
Life-Threatening illness	1.47 ** (1.15–1.88)	1.51 ** (1.17–1.94)	1.45 ** (1.13–1.87)
Life-Threatening Injury	1.38 * (1.06–1.81)	1.40 * (1.06–1.82)	1.39 * (1.06–1.82)
Parent Unavailable	1.54 * (1.09–2.18)	1.52 * (1.07–2.17)	1.60 * (1.12–2.28)
Parent Medical Illness	1.28 * (1.06–1.55)	1.32 ** (1.09–1.60)	1.28 * (1.05–1.56)
Caregiver Physical Abuse	1.40 * (1.07–1.83)	1.50 ** (1.13–1.97)	1.53 ** (1.16–2.01)
**Block 2**			
Negative World Assumptions		0.68 *** (0.62–0.75)	0.69 *** (0.62–0.76)
**Block 3**			
Age			1.01 * (1.00–1.02)
***df***	1, 6	1, 7	1, 8
**χ^2^**	89.09 ***	151.97 ***	157.26 ***

* *p* < 0.05, ** *p* < 0.01, *** *p* < 0.001. The final model correctly classified 65.5% of participants.

## Data Availability

The datasets generated and/or analyzed during the current study are not publicly available to protect the privacy of participants. Interested researchers may reach out to the corresponding author regarding the availability of the data for reproducing the results.
